# Profiling Amino Acid Substitutions in Proteomic Datasets of Alzheimer’s Disease Reveals Biological Significance

**DOI:** 10.3390/biomedicines14102342

**Published:** 2026-10-14

**Authors:** Peize Zhao, Shengzhi Lai, Shuaijian Dai, Chen Zhou, Ning Li, Weichuan Yu

**Affiliations:** 1 Individualized Interdisciplinary Program, Hong Kong University of Science and Technology, Hong Kong, China; pzhaoaj@connect.ust.hk; 2 Department of Electronic and Computer Engineering, Hong Kong University of Science and Technology, Hong Kong, China; slaiad@connect.ust.hk (S.L.); czhouau@connect.ust.hk (C.Z.); 3 Department of Biomedical Engineering, School of Basic Medical Sciences, Central South University, Changsha 410013, China; daishuaijian@csu.edu.cn; 4 Division of Life Science, Hong Kong University of Science and Technology, Hong Kong, China

**Keywords:** amino acid substitution, TMT proteomics, Alzheimer’s disease, peptidoform identification, amino acid substitutomics

## Abstract

**Background**: Amino acid (AA) substitutions in Alzheimer’s disease (AD) have been studied almost exclusively at the DNA and RNA levels, and the proteome-level substitution landscape remains largely uncharacterized. **Methods**: We applied an open-search substitutomics pipeline (PIPI-C with Comet re-scoring, 1% PSM-level FDR) to two independent AD brain proteomic datasets, the AMP-AD superior temporal gyrus cohort (244 individuals) and PXD013753, excluding substitutions whose mass shifts coincide with common post-translational modifications. **Results**: We quantified 789 substitution-bearing peptidoforms from 185 proteins, corresponding to 784 protein-level substitution events. N > M was the most frequent N > X substitution in both datasets. In an institution-stratified case–control meta-analysis, 138 peptidoforms were more and 142 less abundant in AD than in neuropathological controls. Of 774 genome-mappable events, 710 (91.7%) were recorded in neither UniProt nor gnomAD, and only five matched a population variant with allele frequency ≥ 0.1%. The unrestricted search recovered six literature-reported sites in GFAP, ACTG1, and HBB. **Conclusions**: Proteome-level profiling reveals substitution events in AD brains that catalogued germline variation does not explain; their molecular origin requires orthogonal validation.

## 1. Introduction

Alzheimer’s disease (AD) is a major neurodegenerative disorder affecting millions of individuals worldwide. The core clinical features of AD include progressive memory loss, executive dysfunction, and ultimately global cognitive decline [1]. These cognitive deficits are accompanied by prominent histopathological alterations, such as hippocampal atrophy and subsequent neuronal loss [2]. With the ongoing aging of the global population, the global economic cost associated with AD is anticipated to surge to USD 2 trillion by 2030 [1]. Despite extensive research efforts, the etiology of AD remains elusive, and existing treatments only offer symptomatic relief without halting disease progression. A better understanding of the molecular mechanisms of AD is therefore needed to develop more effective treatments.

Amino acid (AA) substitutions in cellular proteins are one possible contributor to AD progression. Substitutions can disrupt native protein structure and function, compromise proteostasis, and reinforce the pathological hallmarks of the disease [3,4,5]. AA substitutions originate from three distinct sources: DNA mutations, mRNA mutations, and errors during protein translation [6]. However, previous research has extensively focused on genomic [7,8,9] and transcriptomic alterations [10,11,12] in AD. In contrast, the majority of AD proteomic studies have focused heavily on post-translational modifications (PTMs) [13,14,15,16]. Only a small fraction of research has targeted AA substitutions. Existing studies on AA substitutions in AD are confined to a small panel of pre-defined variants, leaving the global landscape of proteome-level substitutions largely unmapped. For example, the C410Y and L435F mutations in presenilin-1 (PSEN1), as well as the N141I, T122P, M239V, and M239I mutations in PSEN2, have been shown to alter the ratio of *β*-amyloid protein (A*β*) [17,18]. Large-scale, proteome-level analyses of AA substitutions in AD are therefore lacking.

Identifying AA substitutions in peptides is technically challenging because several substitutions can coexist on one peptide and detection coverage must be balanced against reliability. We therefore used the AA substitutomic search pipeline, which combines open and closed search strategies [19]. Specifically, the AA substitutomic pipeline employs PIPI-C [20] as the primary open-search engine, which is powerful for identifying non-specific AA substitutions, even in cases where multiple substitutions occur on the same peptide. To reduce false discoveries, all candidate substitutions are re-scored using Comet [21], a closed-search engine that requires a pre-specified list of variable modifications. This study aimed to apply this framework to AD brain proteomes to (i) characterize the proteome-level landscape of AA substitutions in the AMP-AD superior temporal gyrus cohort, (ii) determine which substitution events differ in abundance between AD and neuropathological controls, (iii) examine the substitutions carried by AD-associated cytosolic and protein-binding proteins, and (iv) assess whether the observed substitution patterns recur in an independent AD proteomic dataset (PXD013753).

## 2. Methods

### 2.1. AD Proteomic Dataset and Data Preprocessing

For this study, we accessed proteomic data from the AMP-AD consortium via the AD Knowledge Portal (https://www.synapse.org/, accessed on 6 September 2024) [22]. The dataset included samples from the superior temporal gyrus (STG), contributed by 244 individuals across control and pathologically defined AD cases [23]. To validate the generalizability of our findings, we further retrieved an independent validation dataset from ProteomeXchange under the identifier PXD013753 (https://www.ebi.ac.uk/pride/archive/projects/PXD013753, accessed on 19 May 2025) [24], which included samples from 33 individuals. MS2 spectra that have valid contents were selected and converted to MGF format using MSConvert (version 3.0) [25]. We retrieved 1824 raw files from the AMP-AD repository. However, during data preprocessing, one file was excluded due to missing valid MS2 spectra in the resulting MGF file. Consequently, subsequent analyses utilized a total of 1823 MGF files.

### 2.2. AD Proteomic Data Analysis

The FASTA file for human proteins was retrieved from UniProt (https://www.uniprot.org/, accessed on 24 September 2024) [26]. We set the following parameters for PIPI-C (version 1.4.7): an MS1 tolerance of 10 ppm, an MS2 tolerance of 0.01 Da, trypsin as the enzyme allowing up to two missed cleavages, and a peptide length range of 6–50 AAs. Fixed modifications included carbamidomethylation (C, +57.0215 Da) and the isobaric tandem mass tag (TMT) label on lysine. Because the two datasets were labeled with different reagents, this fixed modification was TMTpro (K, +304.2071 Da) for AMP-AD and TMT (K, +229.1629 Da) for PXD013753. Variable modifications encompassed the same reagent on S and the peptide N-terminus (TMTpro for AMP-AD, TMT for PXD013753) and all possible single AA substitutions (using UNIMOD [27]), with a [−250 Da, +250 Da] modification search range. Isoleucine and leucine were regarded as equivalent in this study. Substitutions whose mass shift coincides with that of a common post-translational modification (Table 1) were excluded from all downstream analyses. This exclusion includes N > D and Q > E (+0.98402 Da), which cannot be separated from deamidation or, above a precursor mass of approximately 1.9 kDa at the 10 ppm tolerance, from a +1 Da isotope-peak misassignment (+1.00336 Da); no dedicated isotope-error filter was applied beyond this exclusion. The false discovery rate (FDR) was controlled at 1% at the peptide-spectrum match (PSM) level in both searches. In our previous benchmark on simulated and synthetic peptide datasets, the pipeline showed false-identification rates for substitutions comparable to those of other open-search engines and the highest residue-level localization accuracy when two or three substitutions occur on one peptide; it also recovered the expected increase of the engineered substitutions in a mistranslating-tRNA yeast dataset [19]. Decoy proteins were incorporated to manage the FDR using a target-decoy approach by appending shuffled decoy sequences to the target FASTA, while all other parameters were maintained at their default settings.

In the Comet (version 2024.01 rev. 0) search, we re-searched only those spectra whose PIPI-C identification carried at least one AA substitution and whose substitution-bearing peptidoform had a complete PIPI-C modification profile shared by at least two spectra within the same raw file. For each such spectrum, the per-spectrum modification profile reported by PIPI-C was supplied to Comet as the variable-modification list. All other parameters for the Comet search were consistent with those utilized in PIPI-C. Only PSMs that were identified by both search engines were preserved for bioinformatic analysis. This re-scoring step does not constitute independent validation in the strict sense, since the variable-modification list per spectrum is inherited from PIPI-C; rather, it filters out identifications that fail to reproduce under a different scoring function. Because each spectrum was evaluated only against the modifications proposed for it by PIPI-C, the Comet search space was narrower than that of a conventional variable-modification search; this restriction avoids both the increase in search time and the increase in random matches that accompany large variable-modification spaces.

### 2.3. Quantification Details

Quantification was restricted to PSMs whose TMTpro labeling pattern was consistent with the experimental design. Peptides supported by multiple PSMs were classified as quantifiable. Reporter ion intensities corresponding to each PSM were extracted directly from the MS2. We assumed that all samples contained unregulated peptides with abundances akin to those found in the common reference (CR) sample. Consequently, after normalization, these peptides were expected to yield log ratios centered at zero. To achieve this, we implemented a two-component normalization of TMT label intensities using CR intensities as the reference standard [23].

At the PSM level, log ratios were calculated as the average of log-transformed values across AD patients for each PSM. Peptides with the same modification profiles were combined into unique PTM site patterns (UPSPs). The UPSP-level ratio was calculated by averaging all constituent PSM-level TMT ratios within each subgroup. Then, we derived experiment-wide average log ratios for each UPSP. For downstream analysis, we retained only UPSPs detected in more than 60% of the 19 TMTpro experiments (i.e., in at least 12 of 19 experiments) for quantification. This criterion applies to the AMP-AD dataset only; PXD013753 consists of a single TMT experiment and is therefore not subject to it. For each retained UPSP, statistical significance of the deviation of its experiment-wide average log ratio from zero was assessed using one-sample t-tests across experiments, with Benjamini–Hochberg FDR correction applied to generate adjusted p-values for each UPSP. Because all ratios are computed against the CR channel, an upregulated UPSP throughout this work denotes a UPSP whose abundance in AD samples is significantly higher than that in the common reference, rather than one showing a significant difference between AD and control samples.

The case–control contrast reported in Section 3.2.1 was computed separately. AD and control status follows the harmonized neuropathological classification distributed with the dataset. Reporter intensities were first divided by the total reporter signal of their channel within each experiment, and log ratios were taken against the mean of that experiment’s global internal standard channels. Within each experiment the AD channels and the control channels were averaged and their difference taken, giving one paired estimate per peptidoform per experiment; because the two contributing institutions occupy systematically different reporter channels, this was done separately within Emory and within Mayo Clinic channels. The per-institution mean and standard error across experiments were combined by inverse-variance fixed-effect meta-analysis and referred to a t distribution, with Benjamini–Hochberg correction applied once across the 1678 substitution-bearing peptidoforms testable in both institutions, and events were retained only when the effect had the same sign in both. Between-institution heterogeneity is reported for every event in Supplementary File S1.

### 2.4. Substitution Occupancy

We adapted a biotin occupancy-like metric [28,29]. The N > X substitution occupancy of a protein was defined as the number of asparagine residues at which an N > X substitution other than N > M was detected, divided by the asparagine density of that protein (the number of asparagine residues divided by the protein length). N > M occupancy was defined analogously using only N > M events. This normalization prevents proteins that are simply asparagine-rich from dominating the ranking. Occupancy was computed only for proteins identified in more than 60% of experiments.

### 2.5. GO Enrichment Analysis

We performed Gene Ontology (GO) enrichment analysis on proteins containing quantified UPSPs that met the following criteria: (1) q-value < 0.05 in quantification results, and (2) the presence of AA substitution modifications. The set of proteins identified in this dataset served as the reference background for these analyses, rather than the whole human proteome, so that categories which are intrinsically over-represented among proteins accessible to shotgun proteomics are not spuriously enriched. The background comprised the 3389 leading proteins of all Comet-validated PSMs, of which 3371 carried a Gene Ontology annotation and entered the test. The study set for this analysis was defined before the exclusion of ambiguous mass shifts listed in Table 1 and therefore comprises 187 leading proteins, of which 185 carried an annotation in the detected-proteome background. The complete results under both backgrounds are provided as Supplementary File S2. The statistical significance of the enrichment was evaluated using the two-tailed Fisher’s exact test, incorporating Benjamini–Hochberg FDR correction (q-value < 0.05). All GO enrichment analyses were implemented using GOATOOLS (version 1.6.5) [30] in Python (version 3.10).

### 2.6. Details of Protein–Protein Interaction Network

To investigate functional protein networks, we obtained protein–protein interaction (PPI) data from STRING (version 12.0) [31], which includes interactions from multiple evidence channels, including experimental data, curated databases, text mining, co-expression, genomic context, and phylogenetic profiling. The PPI network was constructed and visualized using Cytoscape (v3.10.2) [32], with proteins represented as nodes and their interactions as edges.

### 2.7. Details of Protein Structure Prediction and Comparison

Alignment and modeling of protein structures were performed using ChimeraX (version 1.10.1). The structure of GAPDH was obtained through cryo-electron microscopy (PDB: 8G17). The crystal structure of ENO1, derived from human sources and expressed in *Escherichia coli*, was used as a reference (PDB: 2PSN). The structure of human ACTG1 was acquired via cryo-electron microscopy at 3.4 Å resolution (PDB: 8DNF), while the cryo-electron microscopy structure of human TUBA1B was sourced from cabbage looper expression (PDB: 5IJ0). For variant structure prediction, we submitted protein sequences with cumulative AA substitutions to AlphaFold3’s web service (AlphaFold Server, https://alphafoldserver.com/, accessed on 28 February 2025), incorporating only the most strongly regulated AA substitution (highest regulation ratio) at each variable site. We acknowledge that cumulative substitution modeling does not represent any single physical proteoform observed in the data; the resulting structures are interpreted as spatial hotspot maps rather than as functional structural predictions. Among the generated structures, we selected the prediction with the highest pLDDT score for structural comparisons.

### 2.8. Cross-Comparison with Population Variant Resources

To assess whether catalogued germline variation could account for the observed events, each of the 784 protein-level substitution events was cross-compared with the gnomAD v4 population variant resource [33]. The UniProt canonical residue position of each event was mapped to its GRCh38 codon coordinates with the EBI Proteins API coordinates service (https://www.ebi.ac.uk/proteins/api/doc/, accessed on 30 September 2026) [34], selecting a mapping on the primary chromosome assembly. All gnomAD v4 variants within each codon were retrieved through the gnomAD GraphQL API (https://gnomad.broadinstitute.org/api, dataset gnomad_r4, accessed on 30 September 2026), and an event was counted as catalogued when a missense variant at the same residue carried the same amino-acid change; isoleucine and leucine were treated as equivalent. Allele frequencies were computed from the combined exome and genome allele counts. Ten events (nine in CAMK2A and one in an immunoglobulin lambda constant region) could not be mapped to the primary assembly and are reported separately. As a positive control, the procedure recovers HBB Leu111Pro as a catalogued variant. The complete results, including codon coordinates, transcript, matched variant and allele counts, are provided as Supplementary File S3; queries were performed in September 2026. Positions refer to the UniProt canonical sequence of the leading protein of each protein group. To flag ambiguous assignments, the substituted sequence of each of the 789 peptidoforms was searched against all reviewed human UniProt sequences, with isoleucine and leucine treated as equivalent; peptidoforms whose substituted sequence is native to another protein are marked in Supplementary File S4.

## 3. Results

### 3.1. AA Substitutomic Workflow and AA Substitutions Landscape of AD Proteome

**Figure 1:**
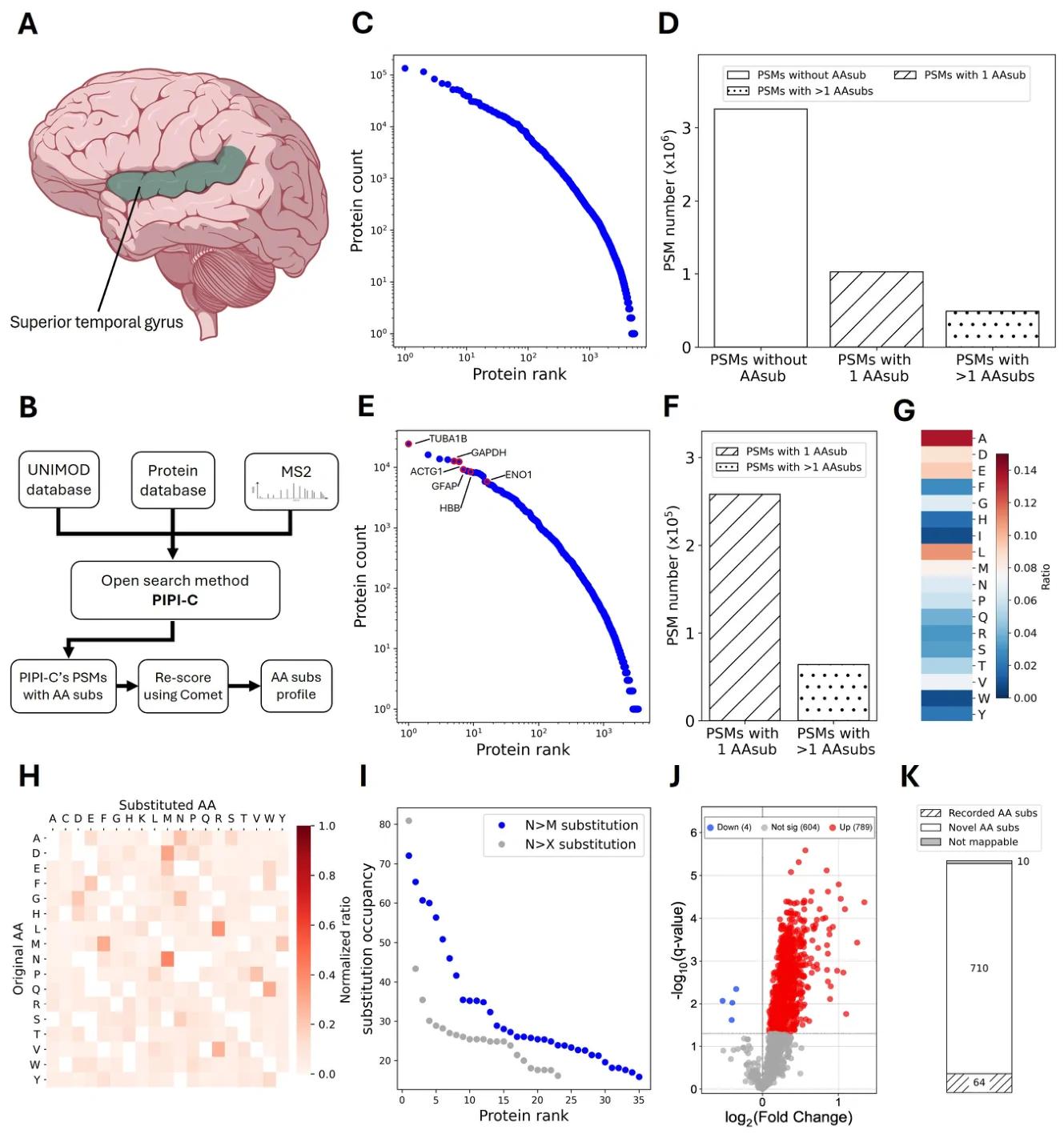
AA substitutomic analysis workflow and AA substitution landscape of AD. (**A**) The schematic representation of the human brain illustrates the location of the STG (green). (**B**) The workflow of proteomic dataset analysis. (**C**) Logarithm–logarithm scale distribution of the protein identification frequency of the PIPI-C-identified PSMs. (**D**) The identification results from PIPI-C. The clean section represents PSMs that lack any AA substitutions, the slashed section indicates PSMs containing a single AA substitution, and the dotted section denotes PSMs that have multiple AA substitutions. (**E**) Logarithm–logarithm scale distribution of the protein identification frequency of the Comet-validated identification results; the six proteins characterized in Section 3.2.2 and Section 3.2.3 are marked in red. (**F**) The validated identification results from Comet. The slashed section indicates PSMs containing a single AA substitution, and the dotted section denotes PSMs that have multiple AA substitutions. (**G**) Normalized ratio of substituted AAs. (**H**) Heatmap displays the normalized ratio for all categories of AA substitutions in validated PSMs within the AMP-AD dataset. The vertical axis represents the original AAs and the horizontal axis denotes the AAs following substitution; each row is normalized to sum to one. Isoleucine and leucine are reported together as L. Data are provided in Supplementary File S5. (**I**) Distribution of the N > M and N > X substitution occupancy in the AMP-AD dataset. Blue dots show the N > M substitution occupancy. Gray dots show the occupancy of N > X substitutions other than N > M. (**J**) The volcano plot illustrates the quantification results for the quantified UPSPs that carry an unambiguous AA substitution. Upregulated UPSPs are marked in red, downregulated UPSPs in blue, and UPSPs without significant regulation in gray. The x-axis shows the log2 ratio of each UPSP relative to the common reference channel, and the dashed horizontal line marks q = 0.05. (**K**) Comparison of the 784 distinct AA substitution events with curated protein variants (UniProt) and population variants (gnomAD). Hatched: events recorded in at least one resource (64); open: events recorded in neither resource (710); gray: events that could not be mapped to the genome (10). Data are provided in Supplementary File S3.

Previous studies have identified disease-relevant AA substitutions in AD-associated proteins [17,18]. Here, we aimed to globally profile AA substitutions in AD from a proteomic standpoint, employing an unrestricted search strategy to capture all potential substitution events. We analyzed proteomic datasets from the AMP-AD consortium [22], encompassing STG brain tissue specimens from 244 individuals across control and pathologically defined AD cases [23] (Figure 1A). All specimens were uniformly homogenized and analyzed via TMT-based mass spectrometry.

The AA substitutomic analytical workflow is outlined in Figure 1B. Spectra were searched with the open-search engine PIPI-C [20], which detects multiple co-occurring modifications on individual peptides [19], using the AMP-AD datasets [22] and the UNIMOD modification and substitution library [27] as input; substitution-bearing spectra were then re-scored with Comet [21], and both engines were controlled at a PSM-level FDR of 1% (Section 2.2). The mass shifts of certain AA substitutions coincide with those of common PTMs; these ambiguous substitutions were excluded from downstream analyses (Table 1).

Figure 1C depicts the protein identification frequency distribution from PIPI-C: most proteins were detected 10 to 1000 times. The relationship between protein identification frequency and rank follows Zipf’s law [35], consistent with the typical distribution of proteomic identifications. Figure 1D quantifies PSMs from PIPI-C, categorized into non-substitution, single-substitution, and multi-substitution groups. After excluding PSMs attributed exclusively to TMT labeling mass shifts, about 31% of PIPI-C-identified PSMs carried a single AA substitution, and 15% harbored multiple substitutions. Retaining multi-substitution peptidoforms is the reason an open search was required here. PIPI-C identified substitution-associated spectra that were then extracted for validation by Comet. All validated PSMs contained at least one AA substitution. Figure 1E shows the protein frequency distribution of validated PSMs from Comet, which also conforms to Zipf’s law. About 20% of validated PSMs contained multiple AA substitutions (Figure 1F). Cysteine carbamidomethylation and lysine TMT labeling were set as fixed modifications, precluding substitution detection on these two residues; thus, substitutions were assessed across 18 AAs.

Substitution frequencies across these 18 residues were non-uniform. Expressed as a fraction of all unambiguous substitution events detected across the dataset, alanine was the most frequently substituted residue (about 14%), while isoleucine and tryptophan were the least frequent (each about 0.8%; Figure 1G). A heatmap of normalized substitution type ratios in the dataset (Figure 1H) revealed that the N > M substitution was the predominant variant among all N > X substitutions, accounting for about 42% of events (Supplementary File S5). In peptides without methionine or tryptophan, which cannot carry an oxidation, N > M remained the most frequent N > X substitution, but its share fell to 19% (13% in the additional dataset), so part of the N > M signal in methionine-containing peptides may reflect oxidation combined with an isotope error. To quantify the relative abundance of N > M substitutions, we calculated the N > M and N > X substitution occupancy for proteins identified in more than 60% of experiments, using the biotin occupancy-like metric [28,29] defined in the Methods section (Figure 1I, Supplementary File S6). N > M substitution occupancy exceeded nearly all other N > X variants, indicating that N > M is detected preferentially over other asparagine substitutions. All raw PIPI-C identification data and validated results are publicly archived in Zenodo [36] with record number 15010408.

We next quantified the differential regulation of UPSPs carrying AA substitutions in AD samples relative to the common reference channel that is included in every TMTpro experiment (Figure 1J, Supplementary File S4). This analysis tests whether the abundance of each UPSP deviates significantly from the common reference across experiments; it is not a direct case–control contrast. Far more UPSPs were elevated relative to the common reference (789) than reduced relative to it (4). The set of 789 UPSPs should be read as substitution events that are reproducibly detected; 94 of them also differ between AD and neuropathological controls in the institution-stratified analysis of Section 3.2.1, and it is that subset for which a disease association is established. These 789 upregulated UPSPs map to 784 distinct protein-level AA substitution events distributed over 185 proteins; identical substitutions detected on different peptidoforms of the same protein were counted once (Supplementary File S7). Each of the 789 peptidoforms was supported by at least 13 PSMs (median 44) and quantified in at least 12 of the 19 experiments, and annotated MS/MS spectra of eight representative substitutions are shown in Supplementary Figure S1. Of these 784 substitution events, 42 (5.4%) overlapped with germline/somatic variants curated in UniProt [26]; the remaining 742 events are not represented in UniProt and therefore correspond to proteome-level observations that may reflect either post-translational events or as-yet-unannotated nucleotide variants (Supplementary File S7). To test whether catalogued population-level germline variation could account for these events, we additionally compared all 784 events with gnomAD v4 (Section 2.8). Of the 774 events that could be mapped to the genome, 40 matched a catalogued missense variant exactly, 18 of which are also UniProt-curated; in total, 64 events are recorded in at least one resource, and 710 (91.7%) are recorded in neither. Moreover, 33 of the 40 gnomAD matches have a population allele frequency below 0.01%, at which fewer than 0.1 carriers would be expected among 244 individuals, and only five events correspond to a variant with an allele frequency of at least 0.1% (Figure 1K, Supplementary File S3). AA substitution profiles varied across proteins: hemoglobin subunit beta (HBB) exhibited the highest normalized substitution ratio, with 22 substitution sites detected in its 147-AA sequence. Actin gamma 1 (ACTG1) carried the highest absolute number of substitution events, with 45 events distributed over 30 sites. In subsequent sections, we focus on significantly regulated UPSPs, with an emphasis on upregulated AA substitutions and proteins harboring multiple substitution sites.

### 3.2. Multiple Significantly Upregulated AA Substitutions in AD-Associated Proteins

To prioritize candidates among the 185 proteins carrying upregulated UPSPs (Supplementary Files S4 and S7), we applied two selection criteria: (1) proteins harboring multiple reproducibly quantified AA substitution events; (2) proteins previously linked to AD in the literature. These criteria yielded six AD-associated proteins. GO enrichment analysis [37] showed that the proteins carrying upregulated substitutions were enriched in cytosol and protein-binding terms, and the six proteins fall into these two categories.

#### 3.2.1. Case–Control Analysis of AA Substitutions in AD Versus Neuropathological Controls

To establish which of these proteins carry substitutions that track AD neuropathology directly, rather than only being reproducibly quantified against the common reference, we performed a case–control analysis using the harmonized neuropathological classification distributed with the AMP-AD dataset. Because every experiment contains both AD and control channels, the contrast was computed within each experiment on identical spectra, separately within Emory and within Mayo Clinic specimens, and the two estimates were combined by inverse-variance fixed-effect meta-analysis (Section 2). Of the 1678 substitution-bearing peptidoforms testable in both institutions, 138 in 52 proteins are more abundant in AD than in neuropathological controls at q < 0.05 with a positive effect in both institutions, and 142 in 75 proteins are less abundant (Supplementary File S1).

The elevated events are markedly concentrated rather than distributed across substituted peptidoforms as a class: the five largest contributors account for half of them; the largest single contributor is a neuron-specific enolase (ENO2), and the remaining four, GFAP, GAPDH, ENO1, and hemoglobin subunit beta (HBB), are among the six proteins characterized below. Taken as a group, the 209 substitution-bearing peptidoforms of the six proteins rank well above the remainder in the AD-minus-control ordering, and this ordering is unchanged when peptidoforms are stratified by reporter-ion intensity. Individually, 91% of the substitution-bearing peptidoforms of GFAP, 92% of those of ENO1, 90% of those of HBB, and 90% of those of GAPDH are more abundant in AD, whereas ACTG1 contributes a single event to the 138 and TUBA1B carried no peptidoform testable in both institutions; both were prioritized on substitution density and structural relevance rather than on differential abundance. These differences reflect the disease-associated abundance of the substituted proteoforms themselves; because reporter-ion ratio compression is intensity-dependent and substitution-bearing peptidoforms are systematically less intense than unmodified ones, the present data do not resolve whether substitution stoichiometry per molecule of protein also changes. For the five proteins testable at the protein level, the AD-control difference of the substitution-bearing PSMs was similar to or smaller than that of the unmodified PSMs of the same protein (for GFAP, 0.30 versus 0.79), and none of the 375 testable proteins showed a larger difference for substitution-bearing PSMs than for unmodified PSMs of matched intensity in both institutions. The disease-associated differences therefore largely follow protein abundance.

#### 3.2.2. AA Substitutomes of Cytosol-Related Proteins in the AD Proteome

Cytosol, also termed the cytoplasmic matrix, is an essential liquid component of eukaryotic cells [38]. The core function of the cytosol is to mediate the transport of metabolites from their biosynthetic sites to their functional utilization sites [39]. Multiple studies have established associations between cytosolic proteins and AD pathogenesis. For instance, Aβ accumulates in the cytosol of lens fiber cells in AD patients [40]. Furthermore, aberrant hyperphosphorylation of the tau protein in the cytosol impairs tau-mediated microtubule stabilization, thereby driving neuronal degeneration and cognitive decline in AD [41].

**Figure 2:**
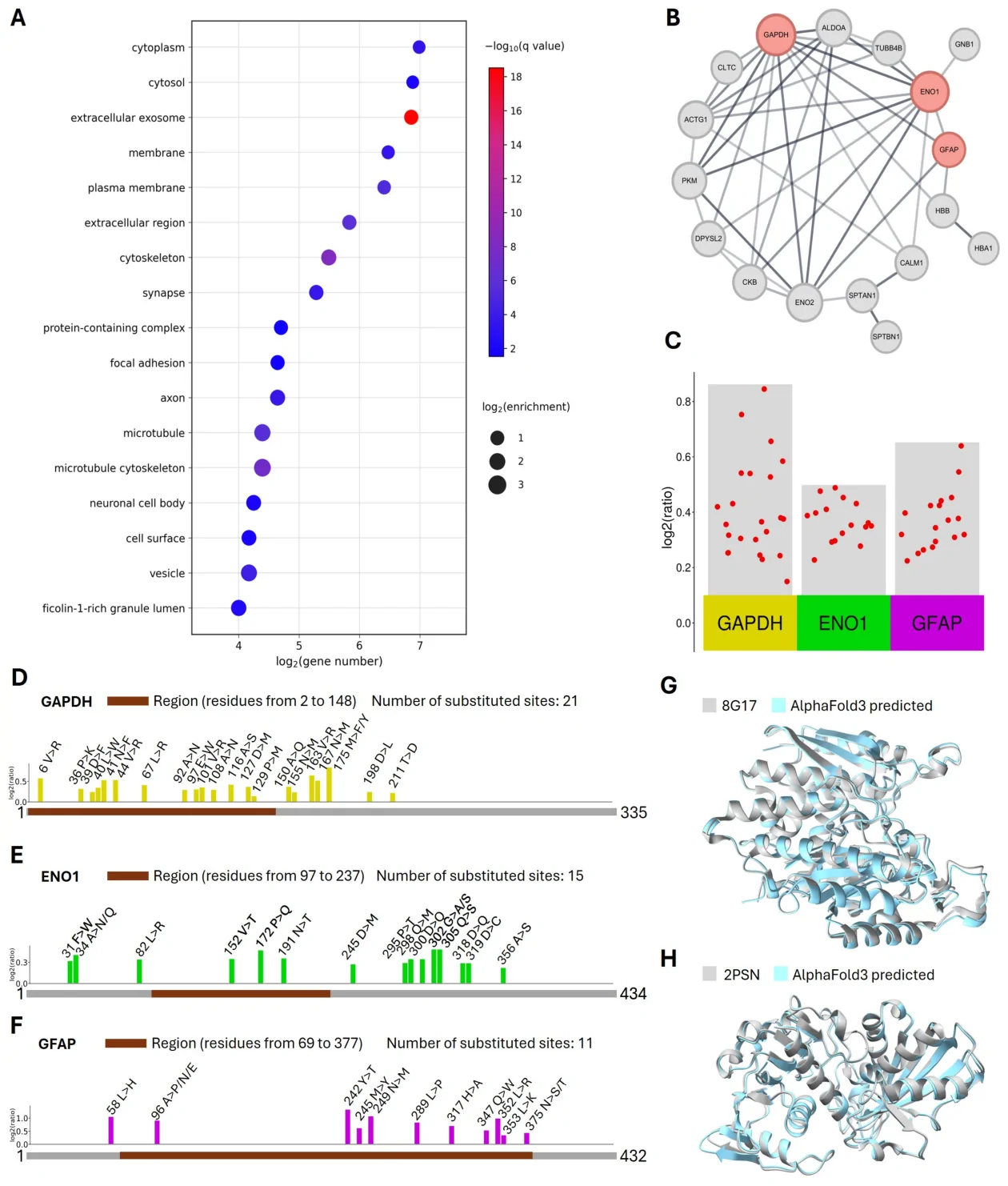
AA substitutomes of cytosol-related proteins in the AD proteome. (**A**) Bubble plot of the GO cellular component enrichment from the upregulated results. The size and color of the bubble represent the enrichment fold of the genes and their minus base 10 logarithm of the q-value, respectively. (**B**) Network of 17 proteins associated with the cytosol, where the size of each node reflects the degree of the respective protein. (**C**) Dot plot of the log2 ratios (relative to the common reference) of the upregulated UPSPs of these proteins. (**D**) Cumulative AA substitution map of GAPDH. Each bar marks a substitution site along the protein sequence; bar height is the log2 ratio of the corresponding upregulated UPSP relative to the common reference, and the label gives the residue position and the observed substitutions. Brown segment: WARS1-binding region (residues 2–148). (**E**) Cumulative AA substitution map of ENO1, drawn as in (D). Brown segment: region required for repression of MYC promoter activity (residues 97–237). (**F**) Cumulative AA substitution map of GFAP, drawn as in (D). Brown segment: intermediate filament rod domain (residues 69–377). (**G**) Structural comparison is conducted between the cryo-electron microscopy structure of human GAPDH (PDB: 8G17) and the structure predicted based on cumulative AA substitutions. The structure of 8G17 is shown in gray, while the AlphaFold3-predicted structure incorporating cumulative AA substitutions is shown in light blue. (**H**) Structural comparison is conducted between the crystal structure of human ENO1 (PDB: 2PSN) and the structure predicted based on cumulative AA substitutions. The structure of 2PSN is shown in gray, while the AlphaFold3-predicted structure incorporating cumulative AA substitutions is shown in light blue.

Among the cellular component terms enriched for proteins carrying upregulated UPSPs, cytosol was one of the most highly represented, encompassing 118 proteins (Figure 2A). The extracellular exosome term was also significantly enriched. Proteins whose substitution-bearing peptidoforms were more abundant in AD than in controls (Section 3.2.1) were likewise enriched in extracellular exosome (36 of 52 proteins, q = 9 × 10^−6^), cytosol, and glycolysis terms, whereas those less abundant in AD were enriched in microtubule and cytoskeletal terms (Supplementary File S2). Figure 2B displays the PPI network of 17 cytosolic proteins identified with at least 10 AA substitutions in the dataset. Node size in the network corresponds to the connectivity degree of each protein. Among these proteins, glyceraldehyde-3-phosphate dehydrogenase (GAPDH) exhibited the highest connectivity degree in the PPI network. GAPDH is a key glycolytic enzyme that catalyzes glucose breakdown to generate energy and carbon metabolites. In addition to its canonical role in aerobic glucose metabolism, emerging evidence has implicated GAPDH in diverse non-metabolic processes. Notably, GAPDH has been reported to interact with core AD-related proteins: Aβ induces disulfide bonding and aggregation of GAPDH in AD brain, and GAPDH aggregates accelerate Aβ amyloidogenesis [42,43].

In addition to GAPDH, two other pivotal proteins, alpha-enolase (ENO1) and glial fibrillary acidic protein (GFAP), are also extensively characterized as AD-associated proteins. Based on our quantitative results, we selected upregulated UPSPs and visualized them in Figure 2C. The specific upregulated AA substitutions identified in these three proteins are detailed in Figure 2D–F. For GAPDH, we mapped 21 AA substitution sites from the AD proteomic dataset (Figure 2D, Supplementary File S4). Notably, at site 175, methionine is substituted by either phenylalanine or tyrosine. Residues 2–148 of GAPDH interact with one isoform of tryptophanyl-tRNA synthetase (WARS1) [44]. Within this region, we identified 14 upregulated substitution sites; whether these substitutions functionally affect WARS1 binding requires biophysical validation.

ENO1, a glycolytic enzyme isoform, catalyzes the conversion of 2-phosphoglycerate to phosphoenolpyruvate [45] and also carries multiple upregulated AA substitutions. ENO1 is a well-documented target of oxidative modification in the brains of AD patients [46] and is detectable across all three pathological stages of AD and in multiple AD models [47,48]. We mapped 15 AA substitution sites in ENO1 from the AD proteome (Figure 2E, Supplementary File S4). Notably, at site 34, alanine is substituted by asparagine or glutamine; at site 302, glycine is substituted by alanine or serine. ENO1 contains a critical functional region (residues 97–237) required for repressing MYC promoter activity [26]. Within this region, we identified three upregulated AA substitutions: V152T, P172Q, and N191T; the N191T peptide is also identical to the native sequence of ENO2. Forced neuronal MYC expression in mice drives cell-cycle re-entry and a neurodegenerative phenotype [49], and phosphorylated c-MYC accumulates in AD and other tauopathies [50]. The three substitutions in the 97–237 region may attenuate the MYC repressive function of ENO1, a possibility that requires functional testing.

GFAP, an astrocytic cytoskeletal protein measurable in peripheral blood, was associated with numerous AA substitutions in our study. It plays an indispensable role in maintaining the mechanical strength and morphological integrity of astrocytes [51]. Recent studies validate GFAP as a reliable indicator of AD pathology, with strong potential for early disease diagnosis [52,53]. We identified 11 upregulated AA substitution sites in GFAP (Figure 2F, Supplementary File S4), all elevated relative to the common reference. GFAP further accounts for 17 of the 138 substitution-bearing peptidoforms that are more abundant in AD than in neuropathological controls in both contributing institutions, and it is the largest contributor under the stricter within-institution criterion (12 of 25 events; Supplementary File S1). Notably, 10 of these substitutions localize to the intermediate filament rod domain (residues 69–377) [26], a region critical for protein structural integrity. Whether these substitutions affect filament assembly has not been tested. At site 96, alanine is replaced by proline, asparagine, or glutamic acid; at site 375, asparagine is substituted by serine or threonine. Additionally, two substitution sites (242 and 352) are associated with Alexander disease, a rare neurodegenerative disorder. The Y242D substitution in GFAP impairs protein oligomerization and solubility by altering residue charge [54]; our identified Y242T substitution alters the residue side chain in a manner that may parallel the documented Y242D effect. The L352P substitution causes severe Alexander disease because proline acts as a helix breaker within the rod domain: its pyrrolidine ring restricts the backbone phi angle, and its imino nitrogen cannot donate the i to i + 4 backbone hydrogen bond that stabilizes the alpha-helix [55]. In our study, we detected an L352R substitution, where hydrophobic leucine is replaced by hydrophilic arginine. This substitution replaces a hydrophobic side chain with a charged one at L352; whether it affects GFAP function remains to be tested.

To evaluate the structural impacts of the identified AA substitutions, we compared the experimental structures of GAPDH [56] and ENO1 [57] with their AlphaFold3-predicted structures harboring cumulative AA substitutions (Figure 2G,H). Because the substitutions may occur on different molecules, these models map the candidate substitution sites onto the structure and are not evidence of structural disruption.

#### 3.2.3. AA Substitutomes of Protein-Binding Proteins in the AD Proteome

Protein binding is crucial in the pathological progression of AD. For instance, the binding affinity of tau to tubulin is regulated by its phosphorylation status, which is tightly modulated by the balanced activity of kinases and phosphatases [58]. Aberrant hyperphosphorylation of tau reduces its tubulin-binding capacity, triggering microtubule disassembly and neuronal dysfunction [59]. Additionally, characterization of the tau-fyn binding interface supports the development of small-molecule inhibitors targeting this interaction, representing a promising therapeutic strategy for AD [60]. Furthermore, studies have confirmed that binding of A*β* and tau oligomers to the amyloid precursor protein (APP) is required for APP internalization into neurons, leading to synaptic dysfunction and memory impairment [61]. Together, these studies link dysregulated protein binding to AD pathogenesis.

**Figure 3:**
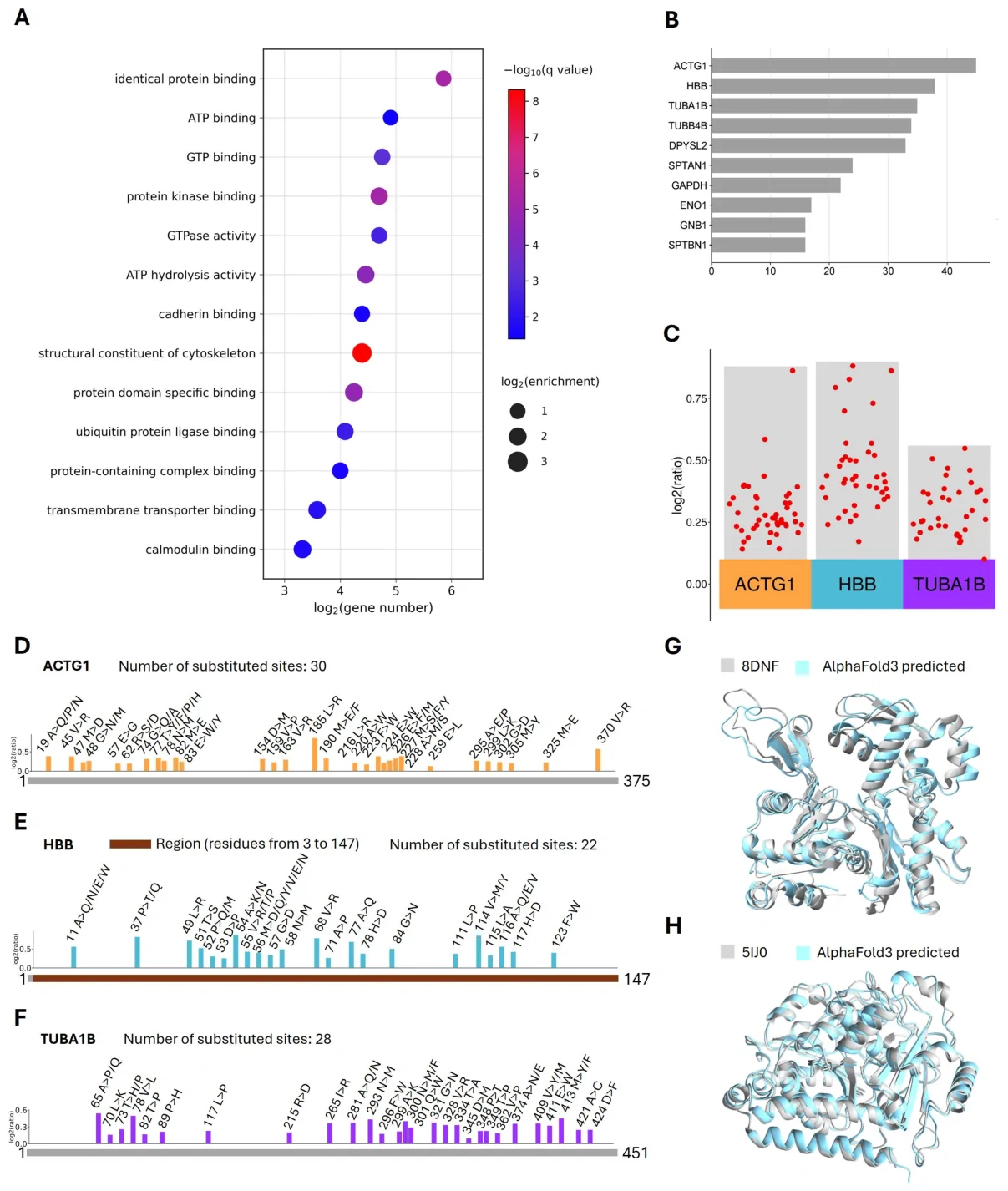
AA substitutomes of protein-binding proteins in the AD proteome. (**A**) Bubble plot of the GO molecular function enrichment from the upregulated results. The size and color of the bubble represent the enrichment fold of the genes and their minus base 10 logarithm of the q-value, respectively. (**B**) Bar plot of the number of identified AA substitution events within the top ten proteins associated with protein binding. (**C**) Dot plot of the log2 ratios (relative to the common reference) of the upregulated UPSPs of these proteins. (**D**) Cumulative AA substitution map of ACTG1. Each bar marks a substitution site along the protein sequence; bar height is the log2 ratio of the corresponding upregulated UPSP relative to the common reference, and the label gives the residue position and the observed substitutions. (**E**) Cumulative AA substitution map of HBB, drawn as in (**D**). Brown segment: globin domain (residues 3–147). (**F**) Cumulative AA substitution map of TUBA1B, drawn as in (**D**). (**G**) Structural comparison is conducted between the cryo-electron microscopy structure of human ACTG1 (PDB: 8DNF) and the structure predicted based on cumulative AA substitutions. The structure of 8DNF is shown in gray, while the AlphaFold3-predicted structure incorporating cumulative AA substitutions is shown in light blue. (**H**) Structural comparison is conducted between the cryo-electron microscopy structure of human TUBA1B (PDB: 5IJ0) and the structure predicted based on cumulative AA substitutions. The structure of 5IJ0 is shown in gray, while the AlphaFold3-predicted structure incorporating cumulative AA substitutions is shown in light blue.

In this study, GO enrichment analysis of the upregulated UPSPs against the identified proteome reveals that the proteins are enriched in a range of specific protein-binding functions (Figure 3A). AA substitutions can alter protein primary structure, modifying protein folding and intra-molecular bonds, ultimately affecting binding sites and functionality [62]. The enrichment of specific binding terms, including identical protein binding, protein kinase binding, and protein domain-specific binding (Supplementary File S2), motivates the AA substitutomic investigation; the generic parent term protein binding is not itself enriched against the identified proteome. Among protein-binding-related proteins, the top three with the highest number of identified AA substitutions are ACTG1, HBB, and tubulin alpha-1B chain (TUBA1B) (Figure 3B). Additionally, based on our quantification results, the upregulated UPSPs of the three proteins that have the most abundant AA substitutions are presented in Figure 3C.

ACTG1 is ubiquitously expressed in most cell types as a core cytoskeletal component that mediates intracellular motility. Emerging evidence has also linked ACTG1 variation to neurotoxicity; for example, carriers of the A allele of the synonymous ACTG1 variant rs1135989 (Ala310) show a markedly increased risk of high-grade vincristine-induced peripheral neurotoxicity [63]. A recent study also reported a positive correlation between ACTG1 expression levels and cognitive scores in AD patients [64]. In our analysis, ACTG1 exhibited the highest number of AA substitutions (45 substitution events distributed over 30 sites; Figure 3D, Supplementary File S7). Notably, 11 residue sites carried alternative AA substitutions, indicating that ACTG1 isoforms with distinct substitution combinations may contribute to AD phenotypic heterogeneity and warrant further investigation. Among these sites, residue 370 is a documented missense mutation locus in ACTG1. Functional assays showed that the V370A mutation impairs cell proliferation under thermal or hyperosmotic stress. Molecular modeling revealed that this mutation disrupts a protein–protein interaction interface in ACTG1, destabilizing the C-terminal tail and cytoskeletal networks [65]. The V370 residue maintains ACTG1 structural integrity via hydrophobic interactions with R116, V134, H371, and C374, interactions conserved across species to stabilize the ACTG1 C-terminus [66,67,68]. The V370R substitution introduces a charged residue at this position; whether it affects C-terminal stability has not been tested. Importantly, V370R shows one of the highest upregulation ratios in ACTG1 UPSPs, suggesting it is among the most consistently detected ACTG1 substitutions in this cohort, and it is the only literature-reported site reproduced with the identical replacement in the additional dataset (Supplementary File S7).

HBB carried 22 distinct AA substitution sites across its 147-residue chain. All substitutions are localized to the highly conserved globin domain (residues 3–147; Figure 3E, Supplementary File S4), which is essential for protein folding and oxygen-binding activity. The clustering of AA substitutions within this critical domain may affect protein stability and molecular interactions. Eight residues (11, 37, 52, 54, 55, 56, 114, and 116) displayed multiple alternative substitutions. Such variability may be relevant to the non-erythroid functions of HBB in neuronal homeostasis [69]. We identified two dense substitution clusters: residues 51–58 and 114–117, with complete substitution coverage of all sites in these regions. Whether these contiguous segments correspond to regions mediating subunit interactions or heme binding remains to be determined.

Of the 22 AA substitution sites identified in HBB, three correspond to positions characterized in the thalassemia literature (111, 115, and 116). Positions are given in UniProt numbering, which retains the initiator methionine and therefore runs one higher than the mature-chain numbering used in that literature. A further seven positions (37, 49, 51, 57, 71, 78 and 114) carry a substitution matching a UniProt-curated variant (Supplementary File S7). The remaining positions are recorded in neither source. Residues 115 and 116 lie within the 114–117 substitution cluster adjacent to site 111. Nucleotide mutations at these positions cause severe thalassemia phenotypes via hemoglobin dysfunction [70,71,72,73]. Of the three, only Leu111Pro is identical to a reported pathogenic variant [74]; at positions 115 and 116, we detect different replacements from those reported. These variants lie in the G helix and destabilize the heme pocket; the sensitivity of globin helices to such alterations is illustrated more generally by the effect of helix truncation on heme affinity and chaperone binding [75]. Some evidence connects thalassemia to AD, including learning and memory deficits in thalassemic patients [76]. A nationwide retrospective cohort study further reported a 1.88-fold increased risk of dementia in thalassemia patients after adjusting for covariates [77]. Whether the substitutions detected at these positions bear on the reported association between hemoglobinopathies and dementia remains to be tested.

TUBA1B, an essential *α*-tubulin isotype governing microtubule dynamics and cytoskeletal integrity, harbored 28 distinct AA substitution sites in our analysis (Figure 3F, Supplementary File S4). Notably, residues 65, 73, 281, 300, 374, 409, and 413 carried multiple alternative substitutions, placing TUBA1B among the proteins with the highest polymorphic substitution density. As a core component of neuronal microtubules, TUBA1B maintains microtubule stability, axonal transport, and neuronal communication, functions severely compromised in AD [78]. Microtubule networks are extensively destabilized in AD, partially due to impaired structural integrity of TUBA1B-containing microtubules, directly driving synaptic degeneration and neuronal apoptosis [79,80,81]. TUBA1B dysfunction is also tightly linked to tau pathology: under physiological conditions, tau stabilizes microtubules via direct binding to TUBA1B, while pathological tau in AD loses this binding affinity, causing defective mitochondrial trafficking, synaptic vesicle dysregulation, and accelerated neurodegeneration [79]. All TUBA1B UPSPs detected here are upregulated relative to the common reference.

To evaluate the structural impacts of AA substitutions on protein-binding proteins, we compared the experimental structures of ACTG1 and TUBA1B with their AlphaFold3-predicted structures harboring cumulative substitutions (Figure 3G,H). As for GAPDH and ENO1, these models map candidate substitution sites, and they are not evidence of structural disruption.

### 3.3. Verification Using One Additional AD Proteomic Dataset

To assess the generalizability of the pipeline and the qualitative recurrence of substitution sites, we analyzed an additional AD dataset obtained from ProteomeXchange [82] under the identifier PXD013753 [24].

**Figure 4:**
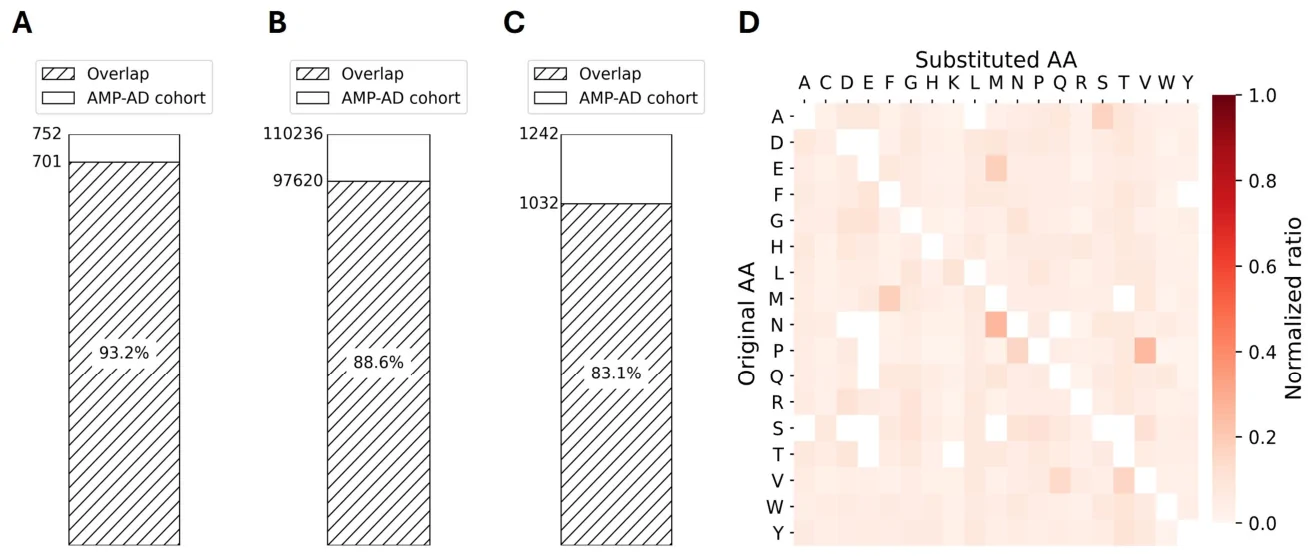
Generalizability of the pipeline in the additional proteomic dataset. (**A**) The overlap results of identified protein numbers. Slashed section: proteins that are replicated in the additional dataset. (**B**) The overlap results of PSM numbers. Slashed section: PSMs with the same peptide sequence in the additional dataset. (**C**) The overlap results of peptide-sequence numbers for the significantly upregulated UPSPs. Slashed section: peptide sequences that are replicated in the additional dataset. (**D**) Heatmap displays the normalized ratios for all categories of AA substitutions in the additional dataset. The vertical axis represents the original AAs, while the horizontal axis denotes the AAs following substitution; each row is normalized to sum to one. Isoleucine and leucine are reported together as L. Data are provided in Supplementary File S8.

Figure 4A illustrates the overlapping proteins detected in the AMP-AD and the additional dataset. In total, 701 out of the 752 proteins that carry a quantified UPSP in the AMP-AD dataset were also identified in the additional dataset, corresponding to an overlap ratio of 93.2%. We next calculated the overlap of PSMs with the same peptide sequence between the two datasets (Figure 4B), where 97,620 of 110,236 PSMs overlapped, yielding an overlap ratio of 88.6%. Furthermore, we evaluated the overlap at the peptide level. The significantly upregulated UPSPs of the AMP-AD dataset correspond to 1242 distinct peptide sequences, of which 1032 were also identified in the additional dataset (Figure 4C), yielding an overlap ratio of 83.1%. Because these three comparisons are matched on protein identity or on the unmodified peptide sequence, they establish that the same proteins and peptides are re-identified in an independent acquisition; replication of the substitution events themselves is assessed at the site level below. AA substitution sites in the six proteins characterized above also recurred in the additional dataset: 57% for GAPDH (12/21), 80% for ENO1 (12/15), 55% for GFAP (6/11), 90% for ACTG1 (27/30), 86% for HBB (19/22), and 96% for TUBA1B (27/28). Because the additional dataset contains many substitution sites per protein, site-level recurrence is partly expected by chance: 103 of the 127 sites recur, against 74 expected. Requiring the identical replacement, 72 of the 171 substitution events recur, against 15 expected (Supplementary File S7). The normalized ratio for each type of AA substitution in the additional dataset is shown as a heatmap in Figure 4D; the pattern is similar to that of the AMP-AD dataset. Consistent with the AMP-AD dataset, among all N > X substitutions, the N > M substitution is identified as the most prevalent, with an approximate ratio of 26% (Supplementary File S8). The search outcomes from the additional dataset have been stored in Zenodo [36] with record number 15753438.

In summary, the substantial overlap ratios observed between the AMP-AD and additional datasets indicate that the substitution events reported here are qualitatively reproducible in an independently acquired AD proteome. Because PXD013753 comprises a single TMT experiment in which 33 individuals were pooled into eight group-level channels, this comparison supports qualitative reproducibility across two proteomic preparations, not independent statistical validation of the AD-associated abundance differences.

## 4. Discussion

We applied the substitutomics framework [19] to mass spectrometry-based proteomic data from the AMP-AD consortium to obtain a proteome-wide map of AA substitutions in AD. The pipeline combines an open search with PIPI-C [20], which places no restriction on the number of substitutions per PSM, with Comet [21] re-scoring, which removes identifications that do not reproduce under a different scoring function.

We observed consistent AA substitution patterns across both the AMP-AD dataset and the dataset of PXD013753, with the N > M substitution representing the most frequent event among all N > X substitutions. This cross-dataset consistency indicates that the N > M enrichment is reproducible across two independently acquired AD proteomes and two different labeling chemistries, rather than an artifact of a single preparation. Translation fidelity influences gene expression and protein function, and ribosomal errors during mRNA translation occur at an estimated stochastic rate of 10^−4^ per codon [83]. Based on these observations, we propose that the high frequency of the N > M substitution may arise from two interrelated factors: (1) enhanced metabolic dependence on methionine in AD cells, a phenomenon documented in previous cancer studies [84]; (2) dysfunction of methionyl-tRNA synthetase in the nervous systems of AD patients [85].

We also detected substitutions at literature-reported positions in AD-associated proteins; their structural and functional consequences remain to be tested. We compared the substitution events with both curated protein variants in UniProt [26] and population-level genomic variants in gnomAD. Only 64 of the 784 events are recorded in either resource, and 710 of the 774 genome-mappable events (91.7%) are recorded in neither; of the 40 events that match a gnomAD variant, 33 do so at an allele frequency below 0.01%, too rare to be expected in a cohort of 244 individuals. Catalogued germline variation can therefore account for only a small minority of the observed events. This does not establish a translational origin: somatic variation, RNA editing and unannotated variants remain possible, and the origin of individual events cannot be assigned from MS data alone and requires orthogonal validation. The same pipeline is applicable to any isobaric-labeled dataset and is not specific to AD.

Recent studies support the view that protein sequences can depart from the genome at a measurable rate. Proteome-wide searches have detected AA substitutions caused by translation errors [86], and peptides produced by alternate RNA decoding are stable and abundant in mammalian cells and tissues [87]. In the brain, an editing defect in alanyl-tRNA synthetase that causes AA misincorporation leads to protein misfolding and neurodegeneration in mice [88], and RNA editing is altered in AD [89]. Many protein-level changes in AD brain are also not observed at the RNA level [90]. These findings make non-genomic substitutions a plausible, though in our data unproven, source of part of the events reported here. Within our data, GFAP Y242T (200 PSMs; more abundant in AD, q = 2 × 10^−7^) and ACTG1 V370R (reproduced with the identical replacement in the additional dataset) combine the strongest identification support with disease association or replication and are the first candidates for experimental validation.

Several limitations of this study should be noted. First, the vast combinatorial complexity of AA substitutions, where any residue can theoretically be replaced by 19 other AAs, creates substantial challenges for mechanistic interpretation. Current functional annotations remain insufficient to fully distinguish causal mechanisms from such complex substitution patterns. Second, the reference-relative quantification establishes which substitution events are reproducibly detected across experiments, whereas the institution-stratified case–control analysis in Section 3.2.1 establishes which of them differ between AD and neuropathological controls; the two answer different questions. The latter resolves a concentrated rather than a comprehensive set: 138 substitution-bearing peptidoforms in 52 proteins, with half of them in five proteins. It is limited by the number of TMTpro experiments available within each institution rather than by the quality of the identifications. Consistent with the institution-associated variance previously documented for this dataset [23], larger cohorts matched within institutions would be expected to resolve more of them. Third, technical constraints of mass spectrometry affect our interpretations: the method captures population-averaged proteomic profiles rather than single-molecule resolution. Thus, the detected substitutions likely occur across different protein molecules rather than simultaneously on the same protein. Our structural modeling places them on a single structure for visualization and cannot determine their co-occurrence. Furthermore, the mass shift of N > M (+16.99756 Da) differs from that of oxidation combined with a ^13^C isotope error (+16.99827 Da) by 0.47 ppm at 1500 Da, below the precursor tolerance used here, so this substitution cannot be distinguished from that combination at the level of individual spectra; its recurrence at high frequency in two independently acquired datasets labeled with different reagents argues against a systematic misassignment but does not exclude it. In addition, 89 of the 789 peptidoforms have a substituted sequence identical to the native sequence of another human protein (for example, HBB T51S matches HBD and ENO1 N191T matches ENO2); these may be peptides of homologous proteins and are flagged in Supplementary File S4. Several substitution classes, such as A > N and L > R (+43.006 and +43.017 Da) and A > Q and G > N (+57.021 Da), are also isobaric or nearly isobaric with carbamylation and carbamidomethylation introduced during sample preparation, so an artefactual origin cannot be excluded for them. Finally, the limited availability of clinical samples and experimental restrictions hinder immediate large-scale wet-lab validation. Determining the role of AA substitutions in AD will therefore require experimental validation of these computational predictions.

## 5. Conclusions

Using the open-search engine PIPI-C [20], we profiled AA substitutions in AD brain proteomes without restricting the analysis to predefined variants. Of the 774 genome-mappable substitution events, 710 (91.7%) are recorded neither in UniProt nor in gnomAD, and only five correspond to a population variant with an allele frequency of at least 0.1%; these events are therefore not explained by catalogued germline variation, although their molecular origin cannot be resolved from MS data alone. The additional dataset supports the qualitative reproducibility of these events, not independent validation of their abundance differences.

We detected substitutions at two GFAP positions (242 and 352) at which mutations causing Alexander disease, a rare neurodegenerative disorder, have been reported. In ACTG1, we detected a substitution at residue 370 (V370R), the position at which the reported V370A mutation impairs cell proliferation under thermal and hyperosmotic stress. Additionally, we detected substitutions at three HBB positions (111, 115, and 116) characterized in the thalassemia literature, of which Leu111Pro is identical to a reported pathogenic variant. While a subset of the identified substitutions is supported by the existing literature, the majority are previously unreported and require experimental validation.

In conclusion, the AA substitutomic pipeline detects proteome-level AA substitutions in AD samples and maps these substitution events to core AD-associated proteins. Of the substitution-bearing peptidoforms testable in both institutions, 138 were more abundant and 142 less abundant in AD than in neuropathological controls; these differences do not by themselves establish a role in pathogenesis but provide candidates for experimental follow-up.

## Figures and Tables

**Table 1:** The ambiguous AA substitutions sharing monoisotopic mass shifts with common PTMs.

AA Site	Monoisotopic Mass	AA Substitution	PTM
Alanine	42.047	A > I or L	Trimethylation
Asparagine	0.984	N > D	Deamidated
Asparagine	14.016	N > Q	Methylation
Asparagine	15.000	N > E	Methylation + Deamidated
Aspartic acid	14.016	D > E	Methylation
Glutamine	0.984	Q > E	Deamidated
Methionine	−29.993	M > T	Met > Hse
Phenylalanine	15.995	F > Y	Oxidation
Proline	31.99	P > E	Dioxidation
Serine	−15.995	S > A	Deoxydation
Serine	14.016	S > T	Methylation
Serine	27.995	S > D	Formylation
Serine	42.011	S > E	Acetylation
Serine	44.008	S > M	Delta:H(4)C(2)O(−1)S(1)
Threonine	27.047	T > K	Ethylamino
Threonine	27.995	T > E	Formylation
C-term	14.016	D > E S > T V > I or L N > Q G > A	Methylation
N-term	14.016	D > E S > T V > I or L N > Q G > A	Methylation
N-term	27.995	S > D T > E	Formylation
N-term	42.011	S > E	Acetylation

## Data Availability

The results of the AMP-AD proteomic dataset are available on Zenodo, with the record number 15010408. https://zenodo.org/records/15010408 (accessed on 12 October 2026). The results of the additional proteomic dataset are available on Zenodo, with the record number 15753438. https://zenodo.org/records/15753438 (accessed on 12 October 2026).

## Supplementary Materials

The following supporting information can be downloaded at: https://www.mdpi.com/article/10.3390/biomedicines14102342/s1. File S1: The 280 substitution-bearing peptidoforms that differ significantly in abundance between AD and neuropathological controls (138 more abundant and 142 less abundant in AD, indicated in the Direction column), with the meta-analyzed effect size and adjusted p-value and the underlying estimates obtained within Emory and within Mayo Clinic. File S2: The complete GO enrichment results for the substitution-bearing proteins under both the whole-genome and the identified-proteome background. File S3: Cross-comparison of the 784 AA substitution events with the gnomAD v4 population variant resource. File S4: The quantification results of the AMP-AD dataset. It contains the AA substitutions, peptide sequence, regulation ratio, and statistics of the results. File S5: The normalized ratio of each AA substitution in the AMP-AD dataset. File S6: The N > M and N > X substitution occupancy in the AMP-AD dataset. File S7: The AA substitution details of all proteins in the AMP-AD dataset, and the AA substitutions that have been recorded in the UniProt database. File S8: The normalized ratio of each AA substitution in the additional AD dataset. Figure S1: Annotated MS/MS spectra of eight representative AA substitution sites.

## References

[1] Tay L.X., Ong S.C., Tay L.J., Ng T., Parumasivam T. (2024). Economic Burden of Alzheimer’s Disease: A Systematic Review. Value Health Reg. Issues.

[2] Scheltens P., Blennow K., Breteler M.M., de Strooper B., Frisoni G.B., Salloway S., Van der Flier W.M. (2016). Alzheimer’s disease. Lancet.

[3] Haass C., Hung A.Y., Selkoe D.J., Teplow D.B. (1994). Mutations associated with a locus for familial Alzheimer’s disease result in alternative processing of amyloid beta-protein precursor. J. Biol. Chem..

[4] Seuma M., Lehner B., Bolognesi B. (2022). An atlas of amyloid aggregation: The impact of substitutions, insertions, deletions and truncations on amyloid beta fibril nucleation. Nat. Commun..

[5] Connelly L., Jang H., Arce F.T., Ramachandran S., Kagan B.L., Nussinov R., Lal R. (2012). Effects of point substitutions on the structure of toxic Alzheimer’s β-amyloid channels: Atomic force microscopy and molecular dynamics simulations. Biochemistry.

[6] Topal M.D., Fresco J.R. (1976). Complementary base pairing and the origin of substitution mutations. Nature.

[7] Seshadri S. (2010). Genome-wide Analysis of Genetic Loci Associated With Alzheimer Disease. JAMA.

[8] Bertram L., Tanzi R.E. (2009). Genome-wide association studies in Alzheimer’s disease. Hum. Mol. Genet..

[9] Rosenberg R.N., Lambracht-Washington D., Yu G., Xia W. (2016). Genomics of Alzheimer Disease: A Review. JAMA Neurol..

[10] Mathys H., Davila-Velderrain J., Peng Z., Gao F., Mohammadi S., Young J.Z., Menon M., He L., Abdurrob F., Jiang X. (2019). Single-cell transcriptomic analysis of Alzheimer’s disease. Nature.

[11] Patel H., Hodges A.K., Curtis C., Lee S.H., Troakes C., Dobson R.J.B., Newhouse S.J. (2019). Transcriptomic analysis of probable asymptomatic and symptomatic alzheimer brains. Brain Behav. Immun..

[12] Patel H., Dobson R.J.B., Newhouse S.J. (2019). A Meta-Analysis of Alzheimer’s Disease Brain Transcriptomic Data. J. Alzheimer’s Dis..

[13] Dujardin S., Commins C., Lathuiliere A., Beerepoot P., Fernandes A.R., Kamath T.V., De Los Santos M.B., Klickstein N., Corjuc D.L., Corjuc B.T. (2020). Tau molecular diversity contributes to clinical heterogeneity in Alzheimer’s disease. Nat. Med..

[14] Abreha M.H., Dammer E.B., Ping L., Zhang T., Duong D.M., Gearing M., Lah J.J., Levey A.I., Seyfried N.T. (2018). Quantitative Analysis of the Brain Ubiquitylome in Alzheimer’s Disease. Proteomics.

[15] Dammer E.B., Na C.H., Xu P., Seyfried N.T., Duong D.M., Cheng D., Gearing M., Rees H., Lah J.J., Levey A.I. (2011). Polyubiquitin linkage profiles in three models of proteolytic stress suggest the etiology of Alzheimer disease. J. Biol. Chem..

[16] Zhang Q., Ma C., Chin L.S., Li L. (2020). Integrative glycoproteomics reveals protein N-glycosylation aberrations and glycoproteomic network alterations in Alzheimer’s disease. Sci. Adv..

[17] Cai Y., An S.S., Kim S. (2015). Mutations in presenilin 2 and its implications in Alzheimer’s disease and other dementia-associated disorders. Clin. Interv. Aging.

[18] Kelleher R.J., Shen J. (2017). Presenilin-1 mutations and Alzheimer’s disease. Proc. Natl. Acad. Sci. USA.

[19] Zhao P., Lai S., Dai S., Zhou C., Li N., Yu W. (2026). Amino Acid Substitutomics: Profiling Amino Acid Substitutions at the Proteomic Scale Unveils Biological Implications and Escape Hypotheses in Cancer. J. Proteome Res..

[20] Lai S., Dai S., Zhao P., Zhou C., Li N., Yu W. (2026). PIPI-C: A Combinatorial Optimization Framework for Identifying Post-translational Modification Hot-spots in Mass Spectrometry Data. Mol. Cell. Proteom..

[21] Eng J.K., Jahan T.A., Hoopmann M.R. (2013). Comet: An open-source MS/MS sequence database search tool. Proteomics.

[22] Hodes R.J., Buckholtz N. (2016). Accelerating Medicines Partnership: Alzheimer’s Disease (AMP-AD) Knowledge Portal Aids Alzheimer’s Drug Discovery through Open Data Sharing. Expert Opin. Ther. Targets.

[23] Seifar F., Fox E.J., Shantaraman A., Liu Y., Dammer E.B., Modeste E., Duong D.M., Yin L., Trautwig A.N., Guo Q. (2024). Large-scale deep proteomic analysis in Alzheimer’s disease brain regions across race and ethnicity. Alzheimer’s Dement..

[24] Hesse R., Hurtado M.L., Jackson R.J., Eaton S.L., Herrmann A.G., Colom-Cadena M., Tzioras M., King D., Rose J., Tulloch J. (2019). Comparative profiling of the synaptic proteome from Alzheimer’s disease patients with focus on the APOE genotype. Acta Neuropathol. Commun..

[25] Chambers M.C., Maclean B., Burke R., Amodei D., Ruderman D.L., Neumann S., Gatto L., Fischer B., Pratt B., Egertson J. (2012). A cross-platform toolkit for mass spectrometry and proteomics. Nat. Biotechnol..

[26] (2019). UniProt Consortium UniProt: A worldwide hub of protein knowledge. Nucleic Acids Res..

[27] Creasy D.M., Cottrell J.S. (2004). Unimod: Protein modifications for mass spectrometry. Proteomics.

[28] Yang N., Ren J., Dai S., Wang K., Leung M., Lu Y., An Y., Burlingame A., Xu S., Wang Z. (2024). The Quantitative Biotinylproteomics Studies Reveal a WInd-Related Kinase 1 (Raf-Like Kinase 36) Functioning as an Early Signaling Component in Wind-Induced Thigmomorphogenesis and Gravitropism. Mol. Cell. Proteom..

[29] Wu K., Yang N., Ren J., Liu S., Wang K., Dai S., Lu Y., An Y., Tian F., Gao Z. (2025). Cytosolic WPRa4 and Plastoskeletal PMI4 Proteins Mediate Touch Response in a Model Organism Arabidopsis. Mol. Cell. Proteom..

[30] Klopfenstein D.V., Zhang L., Pedersen B.S., Ramírez F., Warwick Vesztrocy A., Naldi A., Mungall C.J., Yunes J.M., Botvinnik O., Weigel M. (2018). GOATOOLS: A Python library for Gene Ontology analyses. Sci. Rep..

[31] Szklarczyk D., Kirsch R., Koutrouli M., Nastou K., Mehryary F., Hachilif R., Gable A.L., Fang T., Doncheva N.T., Pyysalo S. (2023). The STRING database in 2023: Protein-protein association networks and functional enrichment analyses for any sequenced genome of interest. Nucleic Acids Res..

[32] Shannon P., Markiel A., Ozier O., Baliga N.S., Wang J.T., Ramage D., Amin N., Schwikowski B., Ideker T. (2003). Cytoscape: A software environment for integrated models of biomolecular interaction networks. Genome Res..

[33] Chen S., Francioli L.C., Goodrich J.K., Collins R.L., Kanai M., Wang Q., Alföldi J., Watts N.A., Vittal C., Gauthier L.D. (2024). A genomic mutational constraint map using variation in 76,156 human genomes. Nature.

[34] Nightingale A., Antunes R., Alpi E., Bursteinas B., Gonzales L., Liu W., Luo J., Qi G., Turner E., Martin M. (2017). The Proteins API: Accessing key integrated protein and genome information. Nucleic Acids Res..

[35] Gabaix X. (1999). Zipf’s Law for Cities: An Explanation. Q. J. Econ..

[36] European Organization for Nuclear Research, OpenAIRE (2013). Zenodo.

[37] Ashburner M., Ball C.A., Blake J.A., Botstein D., Butler H., Cherry J.M., Davis A.P., Dolinski K., Dwight S.S., Eppig J.T. (2000). Gene Ontology: Tool for the unification of biology. Nat. Genet..

[38] Smith A.D., Datta S.P., Smith G.H., Campbell P.N., Bentley R., McKenzie H.A. (2000). Oxford Dictionary of Biochemistry and Molecular Biology.

[39] Verkman A.S. (2002). Solute and macromolecule diffusion in cellular aqueous compartments. Trends Biochem. Sci..

[40] Goldstein L.E., Muffat J.A., Cherny R.A., Moir R.D., Ericsson M.H., Huang X., Mavros C., Coccia J.A., Faget K.Y., Fitch K.A. (2003). Cytosolic β-amyloid deposition and supranuclear cataracts in lenses from people with Alzheimer’s disease. Lancet.

[41] Iqbal K., Alonso A.d.e.l.C., Grundke-Iqbal I. (2008). Cytosolic abnormally hyperphosphorylated tau but not paired helical filaments sequester normal MAPs and inhibit microtubule assembly. J. Alzheimer’s Dis..

[42] Sen T., Saha P., Sen N. (2018). Nitrosylation of GAPDH augments pathological tau acetylation upon exposure to amyloid-β. Sci. Signal..

[43] Itakura M., Nakajima H., Kubo T., Semi Y., Kume S., Higashida S., Kaneshige A., Kuwamura M., Harada N., Kita A. (2015). Glyceraldehyde-3-phosphate Dehydrogenase Aggregates Accelerate Amyloid-β Amyloidogenesis in Alzheimer Disease. J. Biol. Chem..

[44] Wakasugi K., Nakano T., Morishima I. (2005). Oxidative stress-responsive intracellular regulation specific for the angiostatic form of human tryptophanyl-tRNA synthetase. Biochemistry.

[45] Song Y., Luo Q., Long H., Hu Z., Que T., Zhang X., Li Z., Wang G., Yi L., Liu Z. (2014). Alpha-enolase as a potential cancer prognostic marker promotes cell growth, migration, and invasion in glioma. Mol. Cancer.

[46] Shen L., Chen C., Yang A., Chen Y., Liu Q., Ni J. (2015). Redox proteomics identification of specifically carbonylated proteins in the hippocampi of triple transgenic Alzheimer’s disease mice at its earliest pathological stage. J. Proteom..

[47] Sultana R., Robinson R.A., Di Domenico F., Abdul H.M., St Clair D.K., Markesbery W.R., Cai J., Pierce W.M., Butterfield D.A. (2011). Proteomic identification of specifically carbonylated brain proteins in APP(NLh)/APP(NLh) × PS-1(P264L)/PS-1(P264L) human double mutant knock-in mice model of Alzheimer disease as a function of age. J. Proteom..

[48] Opii W.O., Joshi G., Head E., Milgram N.W., Muggenburg B.A., Klein J.B., Pierce W.M., Cotman C.W., Butterfield D.A. (2008). Proteomic identification of brain proteins in the canine model of human aging following a long-term treatment with antioxidants and a program of behavioral enrichment: Relevance to Alzheimer’s disease. Neurobiol. Aging.

[49] Lee H.G., Casadesus G., Nunomura A., Zhu X., Castellani R.J., Richardson S.L., Perry G., Felsher D.W., Petersen R.B., Smith M.A. (2009). The neuronal expression of MYC causes a neurodegenerative phenotype in a novel transgenic mouse. Am. J. Pathol..

[50] Ferrer I., Blanco R., Carmona M., Puig B. (2001). Phosphorylated c-MYC expression in Alzheimer disease, Pick’s disease, progressive supranuclear palsy and corticobasal degeneration. Neuropathol. Appl. Neurobiol..

[51] Cullen D.K., Simon C.M., LaPlaca M.C. (2007). Strain rate-dependent induction of reactive astrogliosis and cell death in three-dimensional neuronal-astrocytic co-cultures. Brain Res..

[52] Kim K.Y., Shin K.Y., Chang K.A. (2023). GFAP as a Potential Biomarker for Alzheimer’s Disease: A Systematic Review and Meta-Analysis. Cells.

[53] Pereira J.B., Janelidze S., Smith R., Mattsson-Carlgren N., Palmqvist S., Teunissen C.E., Zetterberg H., Stomrud E., Ashton N.J., Blennow K. (2021). Plasma GFAP is an early marker of amyloid-β but not tau pathology in Alzheimer’s disease. Brain.

[54] Gorospe J.R., Naidu S., Johnson A.B., Puri V., Raymond G.V., Jenkins S.D., Pedersen R.C., Lewis D., Knowles P., Fernandez R. (2002). Molecular findings in symptomatic and pre-symptomatic Alexander disease patients. Neurology.

[55] Li R., Johnson A.B., Salomons G., Goldman J.E., Naidu S., Quinlan R., Cree B., Ruyle S.Z., Banwell B., D’Hooghe M. (2005). Glial fibrillary acidic protein mutations in infantile, juvenile, and adult forms of Alexander disease. Ann. Neurol..

[56] Choi W., Wu H., Yserentant K., Huang B., Cheng Y. (2023). Efficient tagging of endogenous proteins in human cell lines for structural studies by single-particle cryo-EM. Proc. Natl. Acad. Sci. USA.

[57] Kang H.J., Jung S.K., Kim S.J., Chung S.J. (2008). Structure of human α-enolase (hENO1), a multifunctional glycolytic enzyme. Acta Crystallogr. Sect. D Struct. Biol..

[58] Mandelkow E.M., Biernat J., Drewes G., Gustke N., Trinczek B., Mandelkow E. (1995). Tau domains, phosphorylation, and interactions with microtubules. Neurobiol. Aging.

[59] Avila J. (2008). Tau kinases and phosphatases. J. Cell. Mol. Med..

[60] Lau D.H., Hogseth M., Phillips E.C., O’Neill M.J., Pooler A.M., Noble W., Hanger D.P. (2016). Critical residues involved in tau binding to fyn: Implications for tau phosphorylation in Alzheimer’s disease. Acta Neuropathol. Commun..

[61] Gulisano W., Maugeri D., Baltrons M.A., Fà M., Amato A., Palmeri A., D’Adamio L., Grassi C., Devanand D.P., Honig L.S. (2019). Role of Amyloid-β and Tau Proteins in Alzheimer’s Disease: Confuting the Amyloid Cascade. J. Alzheimer’s Dis..

[62] Villar H.O., Kauvar L.M. (1994). Amino acid preferences at protein binding sites. FEBS Lett..

[63] Ceppi F., Langlois-Pelletier C., Gagné V., Rousseau J., Ciolino C., De Lorenzo S., Kevin K.M., Cijov D., Sallan S.E., Silverman L.B. (2014). Polymorphisms of the vincristine pathway and response to treatment in children with childhood acute lymphoblastic leukemia. Pharmacogenomics.

[64] Li M., Geng R., Li C., Meng F., Zhao H., Liu J., Dai J., Wang X. (2021). Dysregulated gene-associated biomarkers for Alzheimer’s disease and aging. Transl. Neurosci..

[65] Rendtorff N.D., Zhu M., Fagerheim T., Antal T.L., Jones M., Teslovich T.M., Gillanders E.M., Barmada M., Teig E., Trent J.M. (2006). A novel missense mutation in ACTG1 causes dominant deafness in a Norwegian DFNA20/26 family, but ACTG1 mutations are not frequent among families with hereditary hearing impairment. Eur. J. Hum. Genet..

[66] Otterbein L.R., Cosio C., Graceffa P., Dominguez R. (2002). Crystal structures of the vitamin D-binding protein and its complex with actin: Structural basis of the actin-scavenger system. Proc. Natl. Acad. Sci. USA.

[67] Klenchin V.A., Allingham J.S., King R., Tanaka J., Marriott G., Rayment I. (2003). Trisoxazole macrolide toxins mimic the binding of actin-capping proteins to actin. Nat. Struct. Biol..

[68] Hertzog M., van Heijenoort C., Didry D., Gaudier M., Coutant J., Gigant B., Didelot G., Préat T., Knossow M., Guittet E. (2004). The β-thymosin/WH2 domain: Structural basis for the switch from inhibition to promotion of actin assembly. Cell.

[69] Walser M., Hansén A., Svensson P.A., Jernås M., Oscarsson J., Isgaard J., Åberg N.D. (2011). Peripheral administration of bovine GH regulates the expression of cerebrocortical β-globin, GABAB receptor 1, and the Lissencephaly-1 protein (LIS-1) in adult hypophysectomized rats. Growth Horm. IGF Res..

[70] Kobayashi Y., Fukumaki Y., Komatsu N., Ohba Y., Miyaji T., Miura Y. (1987). A novel globin structural mutant, Showa-Yakushiji (β110 Leu-Pro) causing a β-thalassemia phenotype. Blood.

[71] Murru S., Poddie D., Sciarratta G.V., Agosti S., Baffico M., Melevendi C., Pirastu M., Cao A. (1992). A novel β-globin structural mutant, Hb Brescia (β114 Leu-Pro), causing a severe β-thalassemia intermedia phenotype. Hum. Mutat..

[72] de Castro C.M., Devlin B., Fleenor D.E., Lee M.E., Kaufman R.E. (1994). A novel β-globin mutation, β Durham-NC [β114 Leu→Pro], produces a dominant thalassemia-like phenotype. Blood.

[73] Divoky V., Svobodova M., Indrak K., Chrobak L., Molchanova T.P., Huisman T.H. (1993). Hb Hradec Králové (Hb HK) or α2β2 115(G17)Ala→Asp, a severely unstable hemoglobin variant resulting in a dominant β-thalassemia trait in a Czech family. Hemoglobin.

[74] Kazazian H.H., Dowling C.E., Hurwitz R.L., Coleman M., Stopeck A., Adams J.G. (1992). Dominant thalassemia-like phenotypes associated with mutations in exon 3 of the beta-globin gene. Blood.

[75] Domingues-Hamdi E., Vasseur C., Fournier J.B., Marden M.C., Wajcman H., Baudin-Creuza V. (2014). Role of α-globin H helix in the building of tetrameric human hemoglobin: Interaction with α-hemoglobin stabilizing protein (AHSP) and heme molecule. PLoS ONE.

[76] Zafeiriou D.I., Economou M., Athanasiou-Metaxa M. (2006). Neurological complications in β-thalassemia. Brain Dev..

[77] Chen Y.G., Lin T.Y., Chen H.J., Dai M.S., Ho C.L., Kao C.H. (2015). Thalassemia and risk of dementia: A nationwide population-based retrospective cohort study. Eur. J. Intern. Med..

[78] Fu G., Yan S., Khoo C.J., Chao V.C., Liu Z., Mukhi M., Hervas R., Li X.D., Ti S.C. (2023). Integrated regulation of tubulin tyrosination and microtubule stability by human α-tubulin isotypes. Cell Rep..

[79] Santiago-Mujika E., Luthi-Carter R., Giorgini F., Kalaria R.N., Mukaetova-Ladinska E.B. (2021). Tubulin and Tubulin Posttranslational Modifications in Alzheimer’s Disease and Vascular Dementia. Front. Aging Neurosci..

[80] Sferra A., Nicita F., Bertini E. (2020). Microtubule Dysfunction: A Common Feature of Neurodegenerative Diseases. Int. J. Mol. Sci..

[81] Cash A.D., Aliev G., Siedlak S.L., Nunomura A., Fujioka H., Zhu X., Raina A.K., Vinters H.V., Tabaton M., Johnson A.B. (2003). Microtubule reduction in Alzheimer’s disease and aging is independent of tau filament formation. Am. J. Pathol..

[82] Vizcaíno J.A., Deutsch E.W., Wang R., Csordas A., Reisinger F., Ríos D., Dianes J.A., Sun Z., Farrah T., Bandeira N. (2014). ProteomeXchange provides globally coordinated proteomics data submission and dissemination. Nat. Biotechnol..

[83] Shcherbakov D., Teo Y., Boukari H., Cortes-Sanchon A., Mantovani M., Osinnii I., Moore J., Juskeviciene R., Brilkova M., Duscha S. (2019). Ribosomal mistranslation leads to silencing of the unfolded protein response and increased mitochondrial biogenesis. Commun. Biol..

[84] Kaiser P. (2020). Methionine Dependence of Cancer. Biomolecules.

[85] Ognjenović J., Simonović M. (2018). Human aminoacyl-tRNA synthetases in diseases of the nervous system. RNA Biol..

[86] Mordret E., Dahan O., Asraf O., Rak R., Yehonadav A., Barnabas G.D., Cox J., Geiger T., Lindner A.B., Pilpel Y. (2019). Systematic Detection of Amino Acid Substitutions in Proteomes Reveals Mechanistic Basis of Ribosome Errors and Selection for Translation Fidelity. Mol. Cell.

[87] Tsour S., Machné R., Leduc A., Widmer S., Koo E., Guez J., Karczewski K.J., Slavov N. (2026). Alternate RNA Decoding Results in Stable and Abundant Proteins in Mammals. Nature.

[88] Lee J.W., Beebe K., Nangle L.A., Jang J., Longo-Guess C.M., Cook S.A., Davisson M.T., Sundberg J.P., Schimmel P., Ackerman S.L. (2006). Editing-Defective tRNA Synthetase Causes Protein Misfolding and Neurodegeneration. Nature.

[89] Gardner O.K., Wang L., Van Booven D., Whitehead P.L., Hamilton-Nelson K.L., Adams L.D., Starks T.D., Hofmann N.K., Vance J.M., Cuccaro M.L. (2019). RNA Editing Alterations in a Multi-Ethnic Alzheimer Disease Cohort Converge on Immune and Endocytic Molecular Pathways. Hum. Mol. Genet..

[90] Johnson E.C.B., Carter E.K., Dammer E.B., Duong D.M., Gerasimov E.S., Liu Y., Liu J., Betarbet R., Ping L., Yin L. (2022). Large-Scale Deep Multi-Layer Analysis of Alzheimer’s Disease Brain Reveals Strong Proteomic Disease-Related Changes Not Observed at the RNA Level. Nat. Neurosci..

