# Supplementary material for: Profiling Amino Acid Substitutions in Proteomic Datasets of Alzheimer’s Disease Reveals Biological Significance
Source: Biomedicines. 2026 Oct 14;14(10):2342. doi: 10.3390/biomedicines14102342 (PMC13701526; doi:10.3390/biomedicines14102342)
Supplement: Supplementary file 1 [file biomedicines-14-02342-s001.zip › Supplementary_Figure_S1.pdf]

Supplementary note

for

Profiling Amino Acid Substitutions in Proteomic Datasets of Alzheimer's Disease Reveals Biological Significance

Peize Zhao[1,†], Shengzhi Lai[2,†], Shuaijian Dai[3,†], Chen Zhou[2], Ning Li[4,*] and Weichuan Yu[1,2,*]

[1] Individualized Interdisciplinary Program,

Hong Kong University of Science and Technology, Hong Kong, China;

pzhaoaj@connect.ust.hk

[2] Department of Electronic and Computer Engineering,

Hong Kong University of Science and Technology, Hong Kong, China;

slaiad@connect.ust.hk (S.L.); czhouau@connect.ust.hk (C.Z.)

[3] Department of Biomedical Engineering, School of Basic Medical Sciences, Central South University, Changsha 410013, China;

daishuaijian@csu.edu.cn

[4] Division of Life Science,

Hong Kong University of Science and Technology, Hong Kong, China

* Correspondence: boningli@ust.hk (N.L.); eeyu@ust.hk (W.Y.)

† These authors contributed equally to this work.

GAPDH M175F
scan 13608 · mayo_temporaltmt_b5_48

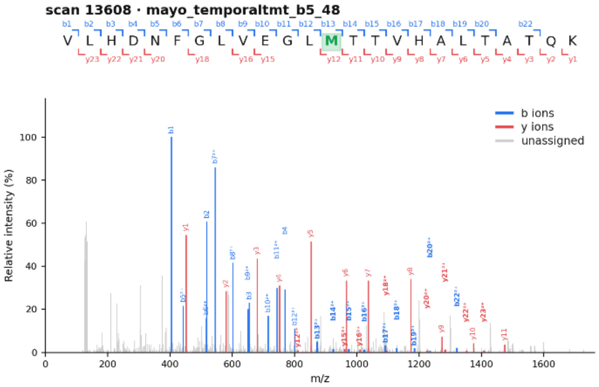

GFAP L352R
scan 10257 · mayo_temporaltmt_b8_67

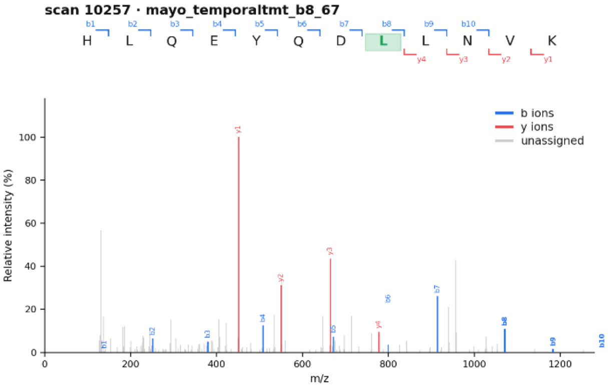

GFAP Y242T
scan 5628 · mayo_temporaltmt_b6_55

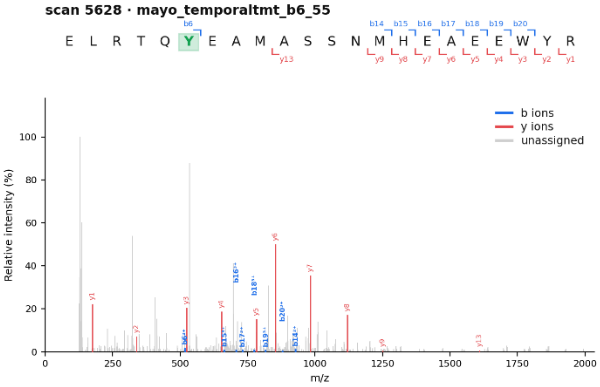

ACTG1 V370R
scan 2946 · mayo_temporaltmt_b16_39

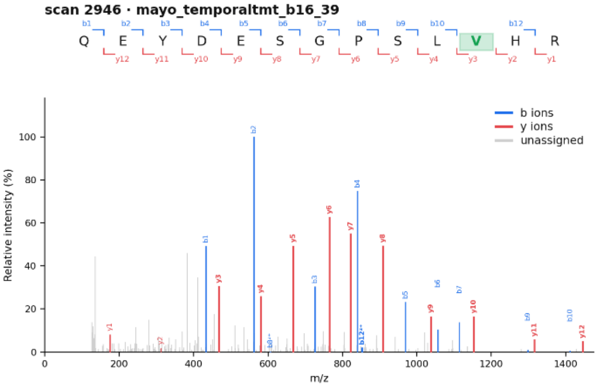

ENO1 V152T
scan 12031 · mayo_temporaltmt_b12_09

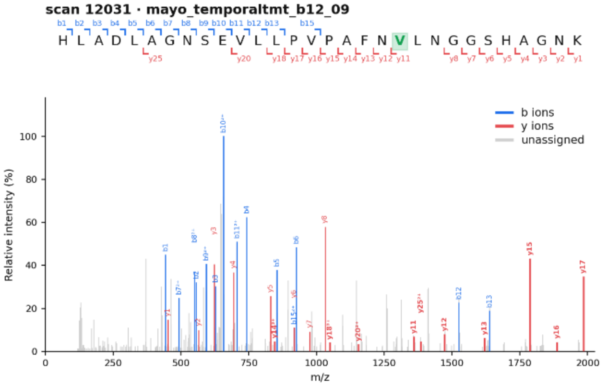

HBB L111P
scan 12590 · mayo_temporaltmt_b4_85

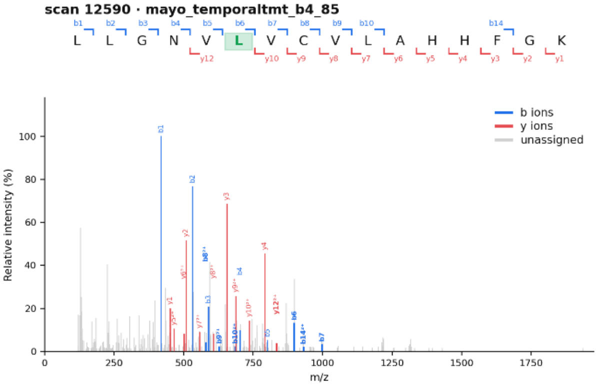

HBB L115A
scan 13257 · mayo_temporaltmt_b18_85

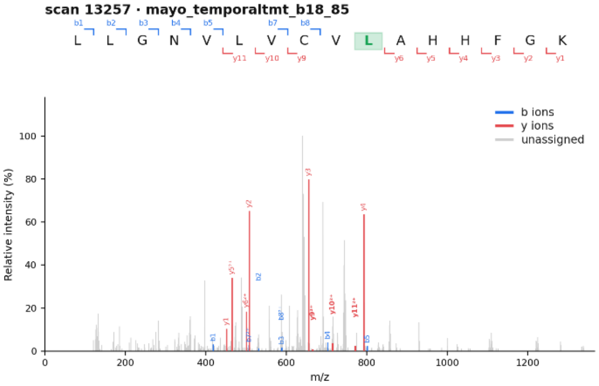

HBB A116Q
scan 12793 · mayo_temporaltmt_b4_83

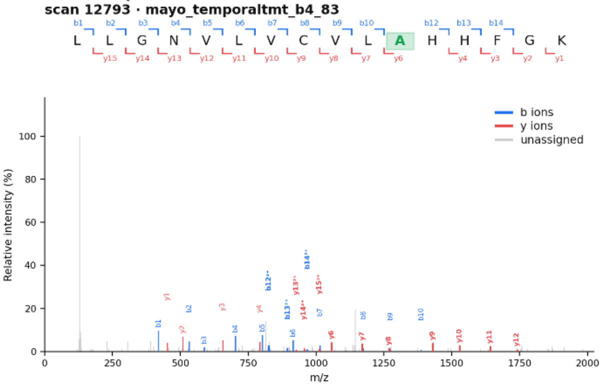

Supplementary Figure S1. Annotated MS/MS spectra of eight representative AA substitution sites.

For each panel, the upper track shows the peptide sequence with the substituted residue highlighted in green and the matched b (blue) and y (red) cleavage positions marked; the lower track shows the annotated spectrum, with matched b and y ions coloured and unassigned peaks in grey. Ion labels in bold denote site-determining ions, whose sequence span includes the substituted residue and which therefore localize the mass shift to a single position. Panel titles give the source raw file and scan number; the corresponding spectra are available in the Zenodo deposit (record 15010408). Raw file names carry the mayo_temporaltmt prefix used for this deposit; every TMT batch contains specimens from both contributing institutions (Emory and Mayo Clinic) and was acquired at Emory.
